# Supplementary material for: Drive the Dirac electrons into Cooper pairs in SrxBi2Se3
Source: Nat Commun. 2017 Feb 15;8:14466. doi: 10.1038/ncomms14466 (PMC5316857; doi:10.1038/ncomms14466)
Supplement: Supplementary Information — Supplementary Figures 1-9, Supplementary Tables 1-2, Supplementary Notes 1-5 and Supplementary References [file ncomms14466-s1.pdf]

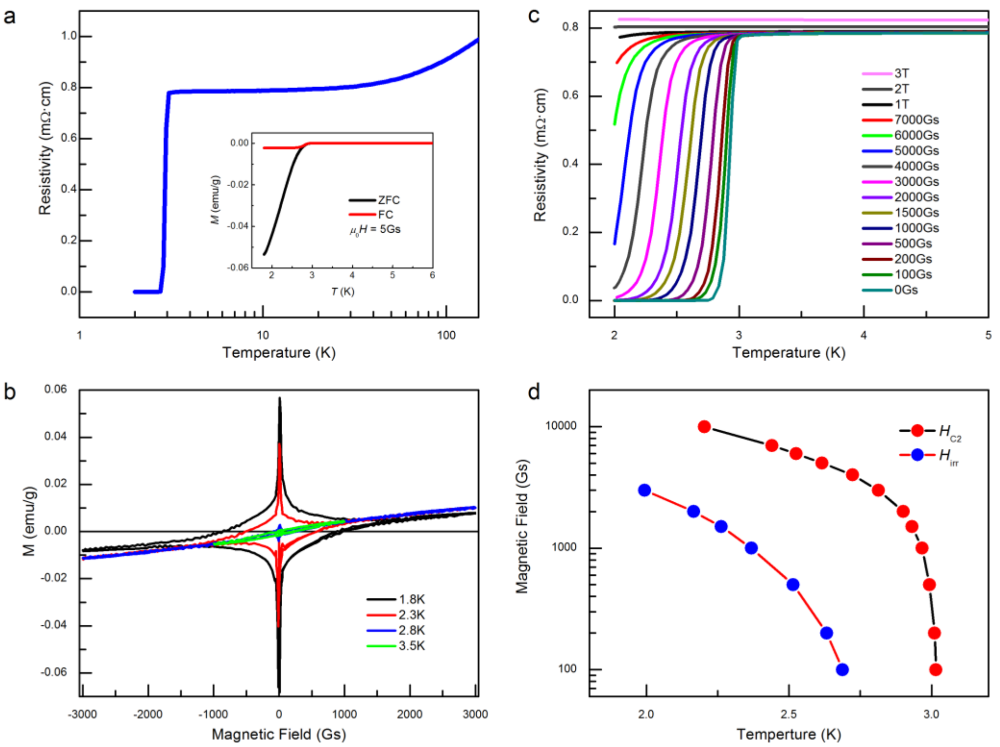

Supplementary Figure 1 | Physical characterization of the $Sr_xBi_2Se_3$ single crystal. a, Temperature dependence of resistivity of a $Sr_xBi_2Se_3$ single crystal at zero field. The inset shows the temperature dependence of magnetic susceptibility measured with zero-field-cooled (ZFC) and field-cooled (FC) processes at an applied magnetic field of 5 Gs. The superconducting transition temperature is about 3 K. The calculated magnetic screening volume is about 91.6% at 1.8 K (without considering the demagnetization factor). **b**, The *M*-*H* loop as a function of temperature with the magnetic field applied parallel to *c*-axis. **c**, The resistivity as a function of magnetic field applied parallel to *c*-axis. **d**, Phase diagram of the irreversibility magnetic field H_{irr} and upper critical field H_{c2} obtained by 1% and 99% of the normal state resistivity ρ_n. The value of H_{c2} at zero temperature is estimated to be about 3.5 T.

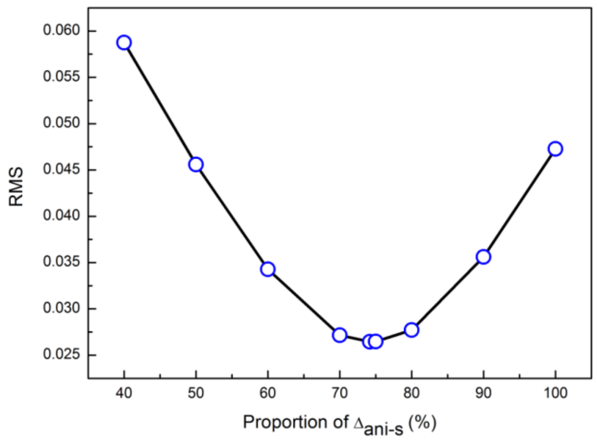

Supplementary Figure 2 | Error of fitting to the STS by tuning the fraction of the ani-*s* wave. The vertical coordinate represents the root-mean-square (RMS) value of the difference between raw data and the fitting results. The smaller the RMS is, the better fitting is assumed. The proportions of 26% (for the *s* wave) and 74% (for the ani-*s* wave) turns out to give the best fit.

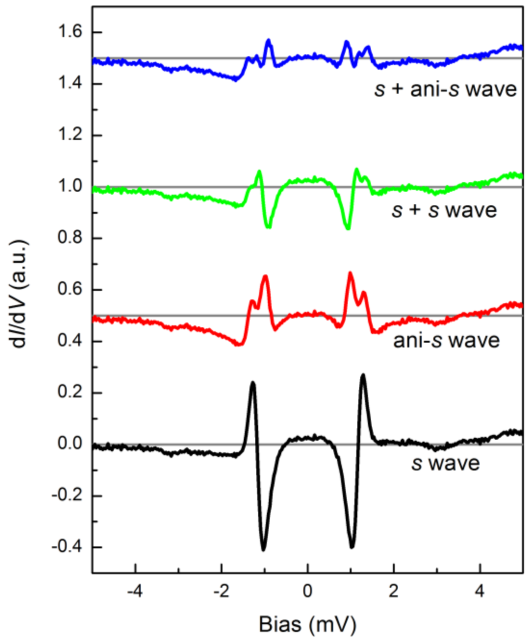

Supplementary Figure 3 | The difference curves between the fitting curves and the raw spectrum. The measured spectrum is fitted by Dynes model with several different superconducting gap functions. The difference curves are offset for clarity. One can find that the fitting with two components associating with double gaps (*s* + ani-*s* wave) can describe experimental data very well.

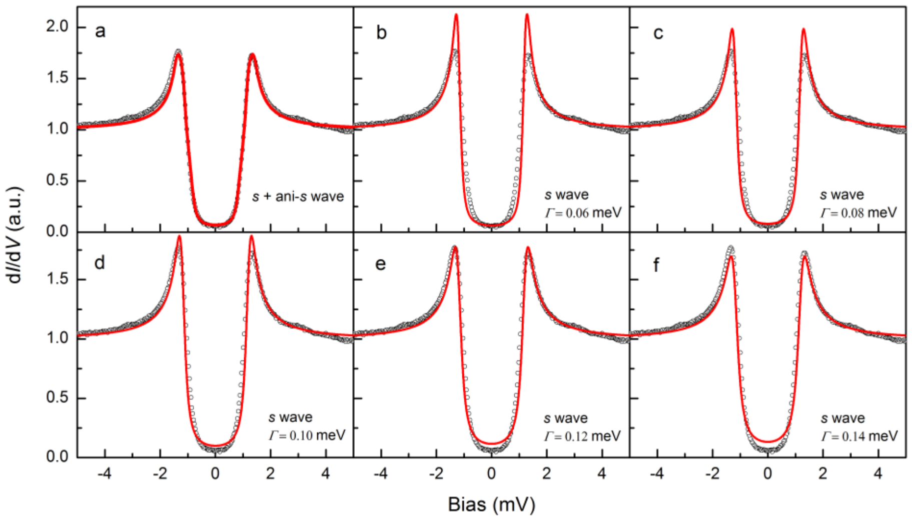

Supplementary Figure 4 | Comparison of the fitting results with different gaps and parameters. a, The fitting curve of the two-component model (*s* + ani-*s* wave) and the raw data (symbols). **b**-**f**, The fitting curves of the single *s* wave model with different *Γ* values.

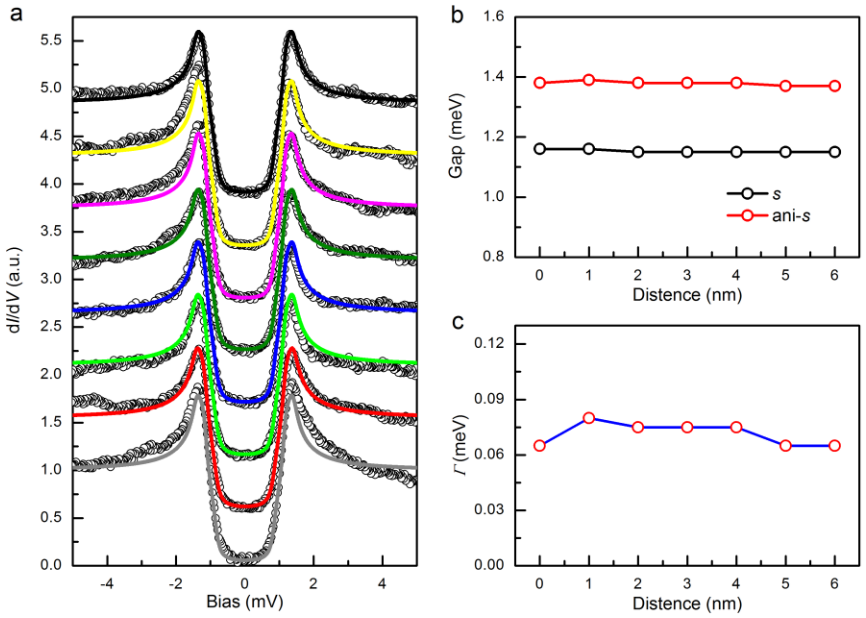

Supplementary Figure 5 | The two-component model (*s* + ani-*s* wave) fitting to the spatially resolved tunneling spectra. a, The theoretical fitting to the spatially resolved tunneling spectra using the two-component model (*s* + ani-*s* wave). The raw data are shown by dots and the fitting results are shown by solid curves. **b**, The spatial variation of gap values of the *s* wave component and the ani-*s* wave component obtained by fitting. One can find that the superconductivity is almost homogeneous in this region. **c**, The spatial variation of *Γ* value obtained by fitting. The horizontal axis in **b** and **c** represents the locations where the spectra were taken.

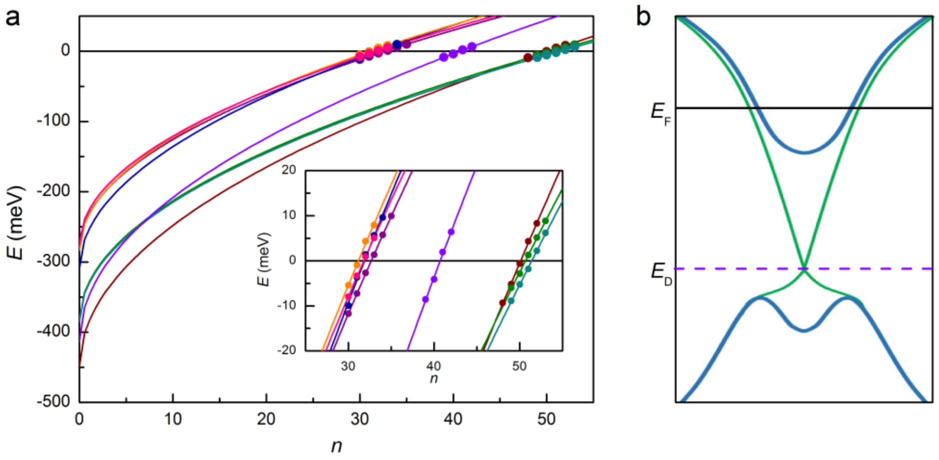

Supplementary Figure 6 | Theoretical fittings to LLs peak positions at 5 T. a, The dots represent the LL peak energies obtained from the STS measured at 5 T. The solid curves are the fitting results. The *n* value here represents the nth LL. The inset shows the zoom-in view of the main panel. **b,** Schematic illustration of the band structure of $Sr_xBi_2Se_3$. The topological SSs are indicated by band marked with the green curves. E_F and E_D represent the Fermi energy and the Dirac point energy.

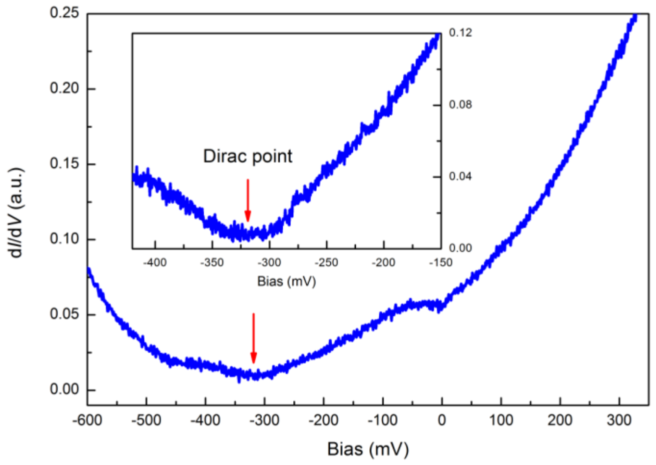

Supplementary Figure 7 | Detection of the Dirac point. The main panel shows the spectrum with the bias range from -600 mV to 350 mV. The inset shows the spectrum measured in the range from -450 mV to -150 mV. Both the spectra are taken at 400 mK and 0 T in an area with few substituted Sr impurities, I_t = 102 pA. The two linear parts of the spectrum in the inset result from the linearly dispersing Dirac cone of the surface states. The Dirac point can be determined to locate at -320 mV as indicated by the red arrows. This value is quite close to -340 mV obtained by fitting to the LLs, which validates the method of determining E_D in the main text.

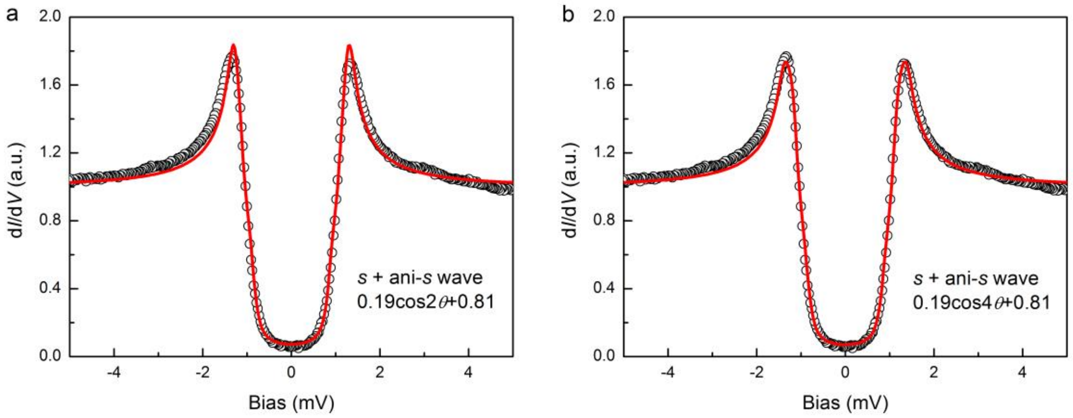

Supplementary Figure 8 | Fitting to the Dynes model with two components (*s* + ani-*s* wave gaps). The gap functions of the ani-*s* wave component in the fitting are displayed in each figure. Other fitting parameters are detailed in Supplementary Note 4.

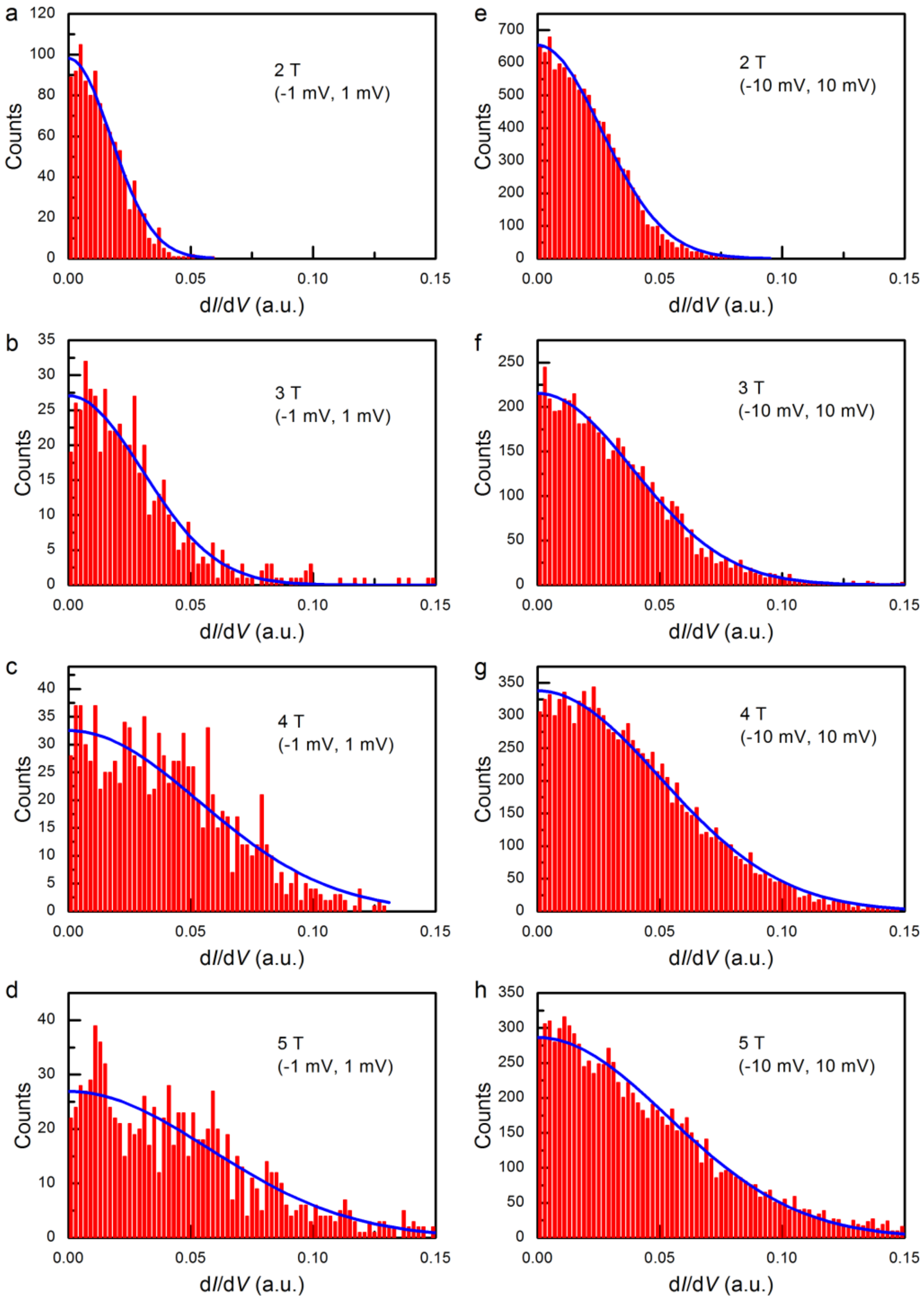

Supplementary Figure 9 | Statistics on the d*I*/d*V* difference and the Gaussian fitting. a-d(**e-h**), The statistics on the absolute values of the differences between the normalized spectrum at different locations and the averaged curve in the region from -1 mV to 1 mV (-10 mV to 10 mV) at different magnetic fields are shown by histograms, and the Gaussian fitting results are plotted using blue lines. The incremental block of d*I*/d*V* for statistics is 0.002.

	s wave	ani-s wave	$s_1 + s_2$ wave	s+ ani-s wave
Standard Deviation	0.09284	0.04602	0.03958	0.02921

Supplementary Table 1 | The standard deviations of the difference value between the raw spectrum and the fitting curves of four different models.

	(-1 mV, 1 mV)			(-10 mV, 10 mV)		
	$\bar{m}_1$	$\bar{x}$	$\lvert\bar{m}_1 - \bar{x}\rvert$	$\bar{m}_{10}$	$\bar{x}$	$\lvert\bar{m}_{10} - \bar{x}\rvert$
2 T	0.01363	0.01385	0.00023	0.02011	0.02071	0.0006
3 T	0.02708	0.02405	0.0030	0.03135	0.03097	0.00039
4 T	0.03957	0.04272	0.00315	0.03919	0.03994	0.00075
5 T	0.04843	0.04603	0.0024	0.04645	0.04237	0.00408

Supplementary Table 2 | Calculation of error bars of the mean values at different fields. $\bar{m}_1$ and $\bar{m}_{10}$ are the mean values calculated from the experimental data by averaging, while $\bar{x}$ is the mean value obtained by the Gaussian functions (see Supplementary Note 5). The error bars are defined as $|\bar{m}_1 - \bar{x}|$ and $|\bar{m}_{10} - \bar{x}|$.

Supplementary Note 1 | Superconducting spectrum fitting by Dynes model.

The spectrum in Fig. 2c is fitted with tunneling current for one gap[1] which reads as

$$I(V) \propto \int_{-\infty}^{\infty} \mathrm{d}\varepsilon \int_0^{2\pi} \mathrm{d}\theta [f(\varepsilon) - f(\varepsilon + eV)] \times \mathrm{Re}\left\{\frac{\varepsilon + eV - i\Gamma}{[(\varepsilon + eV - i\Gamma)^2 - \Delta^2(\theta)]^{1/2}}\right\},$$

where Γ is the broadening parameter, $f(\varepsilon)$ is the Fermi distribution function which contains the thermal broadening effect at finite temperatures. The fitting results with different gap functions are shown in Fig. 2d. The single s-wave fitting to the spectrum yields a superconducting gap value of Δ = 1.2 meV and broadening parameter of Γ = 0.10 meV. For a single anisotropic s-wave gap, the fitting leads to Δ = 1.3 meV and Γ = 0.07 meV. For the two-component fitting with double s-wave gaps, the two mixed components have the proportion of 40%Δ_1 + 60%Δ_2 with Δ_1 = 1.3 meV, Γ_1 = 0.09 meV, Δ_2 = 1.05 meV, Γ_2 = 0.09 meV. For the case of s + ani-s wave, the two components have the proportion of 26% for s wave and 74% for ani-s wave with Δ_s = 1.15 meV, Γ_s = 0.075 meV, $\Delta_{\mathrm{ani}-s} = 1.37(0.19\cos 4\theta + 0.81)$ (meV), $\Gamma_{\mathrm{ani}-s}$ = 0.075 meV. The proportions of 26% (for the s wave) and 74% (for the ani-s wave) are obtained through searching the minimum of the lowest root-mean-square (RMS), as shown in Supplementary Fig. 2.

All the parameters used for fitting are obtained by minimizing the difference between fitting curve and raw data. The fitting curves shown in Fig. 2d are the optimized ones for each model. We further calculated the difference value between the raw spectrum in Fig. 2c and

the fitting curves, and the results are shown in Supplementary Fig. 3. We also calculate the standard deviations for each model, shown in Supplementary Table 1. The fitting with two components (*s* and ani-*s* wave gaps) has the minimum standard deviation and is the best fit among the four models. The fitting curves with a single component of ani-*s*, and *s*+*s* have relatively larger deviations from the raw data in the bias range of 0.7~1.2 mV as shown in Fig. 2d and Supplementary Fig. 3.

For the single *s* wave model, one would have thought that using a larger value of *Γ* will improve the fit by reducing the height of the coherence peak and bringing more density of states into the gapped region and make the single *s* wave model the best description of the spectrum. In Supplementary Fig. 4 we show the comparison of the results of two-component model (*s* + ani-*s* wave) fitting and single *s* wave fitting using different *Γ* values. The raw data In Supplementary Fig. 4 is the same as the spectrum in Fig. 2c. We can see that with increasing *Γ* value, the coherence peaks become less sharp and the in-gap DOS get larger. The single *s* wave fitting cannot track the low energy feature and the coherence peaks at the same time. By comparing with the two-component model (*s* + ani-*s* wave) fitting in Supplementary Fig. 4a, we can conclude that the two-component model fitting can better represent the nature of superconductivity.

In addition, we have also done the two-component model (*s* + ani-*s* wave) fitting to each spatially resolved tunneling spectra in Fig. 2b. The raw data and the fitting results are shown by dots and solid curves in Supplementary Fig. 5a. According to the fitting results, the superconductivity is almost homogeneous in this region.

Supplementary Note 2 | Fitting results to the LL peak positions.

We obtained the bias values of the LL peaks from the spectrum in Fig. 3d and some of the spectra with obvious LL fluctuations in Fig. 4e. The peak positions are fitted with the nth LL peak energies (E_n) by using the equation,

$$E_n = E_{\mathrm{D}} + v_{\mathrm{F}}\sqrt{2eB\hbar|n|},$$

where v_{F} is the Fermi velocity and B = 5 T is the magnetic field. The fitting results are displayed in Supplementary Fig. 6. One can find that there is a finite scattering among the fitting curves. Except for the influence of the spatially local alteration to the electronic states, the scattering can also be induced by the experimental errors. This is because the Landau level peaks as shown in Fig. 4e do not have a regular shape and are always accompanied by other fine structures. So, it is very hard to obtain the accurate bias value of the peaks in Fig. 4e and finite deviation is inevitable. The Landau level index *n* is found to be about 30-50 in the fitting which is very big, so the value of $E_n - E_{n-1} \propto \sqrt{n} - \sqrt{n-1}$ is very small. This also makes the fitting results rather scattered. Although there is uncertainty in the fitting results shown in Supplementary Fig. 6, we can still get the approximate values of E_{D} and v_{F} by averaging the

fitting parameters of different sets of data to lower down the uncertainty, *i.e.*, E_{D} is about -340 meV and v_{F} is about 6.7×10^5 m/s.

In addition, we succeed in detecting the Dirac point by STS in an area with few intercalated Sr impurities at zero magnetic field. The spectrum is shown in the main panel of Supplementary Fig. 7. The inset of Supplementary Fig. 7 shows the spectrum within a smaller bias range. The Dirac point locates at about -320 mV as indicated by the red arrows. The value of -320 mV is comparable to the theoretical fitting to the Landau levels that $E_{\mathrm{D}} = -340$ meV. The d*I*/d*V* value at -320 mV is almost zero which also consists with the property of DOS of the Dirac point.

Supplementary Note 3 | Data treating and normalizing process to the spectra with LL oscillations.

To visualize the effect of the LLs on the spectrum, a proper way to normalize the spectra in Fig. 4 is very important. Usually, the STS measured at zero field in the same area at a high energy outside the gap is smooth and has little difference, for example, one can see the data in Fig. 4a. It is easy to normalize all the curves by selecting the d*I*/d*V* value at a certain voltage as a reference. While because of the oscillations due to the LLs at a finite magnetic field, this becomes nontrivial, because the curve at a energy higher than the gap fluctuates a lot, which makes it difficult to decide at which point all the curves should be normalized. We have tried several schemes and developed an efficient one as addressed below.

For a fixed magnetic field, we first calculate the integral of the spectrum measured at each location between -10 mV to 10 mV, then normalize these spectra measured at different spatial locations by taking the integral as the normalizing factor. Then we obtain the average spectra at each magnetic field. Finally all the spectra at the same field are normalized to unity by dividing the value of d*I*/d*V* on the averaged spectrum at the bias voltage of 10 mV.

Supplementary Note 4 | Dynes model fitting with different anisotropic gap functions.

In fitting the data to the Dynes model with two components (*s* + ani-*s* wave gaps), we used two different gap equations for the ani-*s* wave gap, *i.e.*, 0.19cos4θ +0.81 and 0.35cos2θ +0.65, and the first one seems a little better as shown in Supplementary Fig. 8. However, if merely judging from the quality of fitting, we could not rule out the 2-fold gap function.

Supplementary Note 5 | Method to estimate the error bars of mean values.

The distribution of d*I*/d*V* values of the LL oscillations in the windows of (-1 mV, 1 mV) and (-10 mV, 10 mV) in Fig. 4k-4o are shown in Supplementary Fig. 9, which can be well described by the Gaussian function. We use the general Gaussian function $g(x)$ to fit each distribution and calculate the mean value $\bar{x}$ through

$$\bar{x} = \frac{\int_0^{+\infty} x g(x) \mathrm{d}x}{\int_0^{+\infty} g(x) \mathrm{d}x}.$$

Here x denotes the dI/dV values. The mean value $\bar{x}$ represents the averaged dI/dV value calculated through fitting to the Gaussian function. We then use the absolute difference between $\bar{x}$ and the corresponding mean values shown in Fig. 5a to calculate the error bars, and all the results at different fields are shown in Supplementary Table 2. Those error bars are displayed in Fig. 5a.

The error bars in Fig. 5b are calculated using the function below,

$$\Delta r = \frac{1}{\bar{m}_{10}} \Delta \bar{m}_1 - \frac{\bar{m}_1}{\bar{m}_{10}^2} \Delta \bar{m}_{10},$$

where $\bar{m}_1$ and $\bar{m}_{10}$ are the mean values of the data in the window of (-1 mV, 1 mV) and (-10 mV, 10 mV) shown in Fig. 5a, Δr, $\Delta \bar{m}_1$ and $\Delta \bar{m}_{10}$ are the error values of the ratio value, m_1 and m_{10}, respectively. This is standard way to get the error bar between two divided quantities.

Supplementary References

1. Dynes, R. C., Garno, J. P., Hertel, G. B. & Orlando, T. P. Tunneling Study of Superconductivity near the Metal-Insulator Transition. *Phys. Rev. Lett.* **53**, 2437-2440 (1984).
